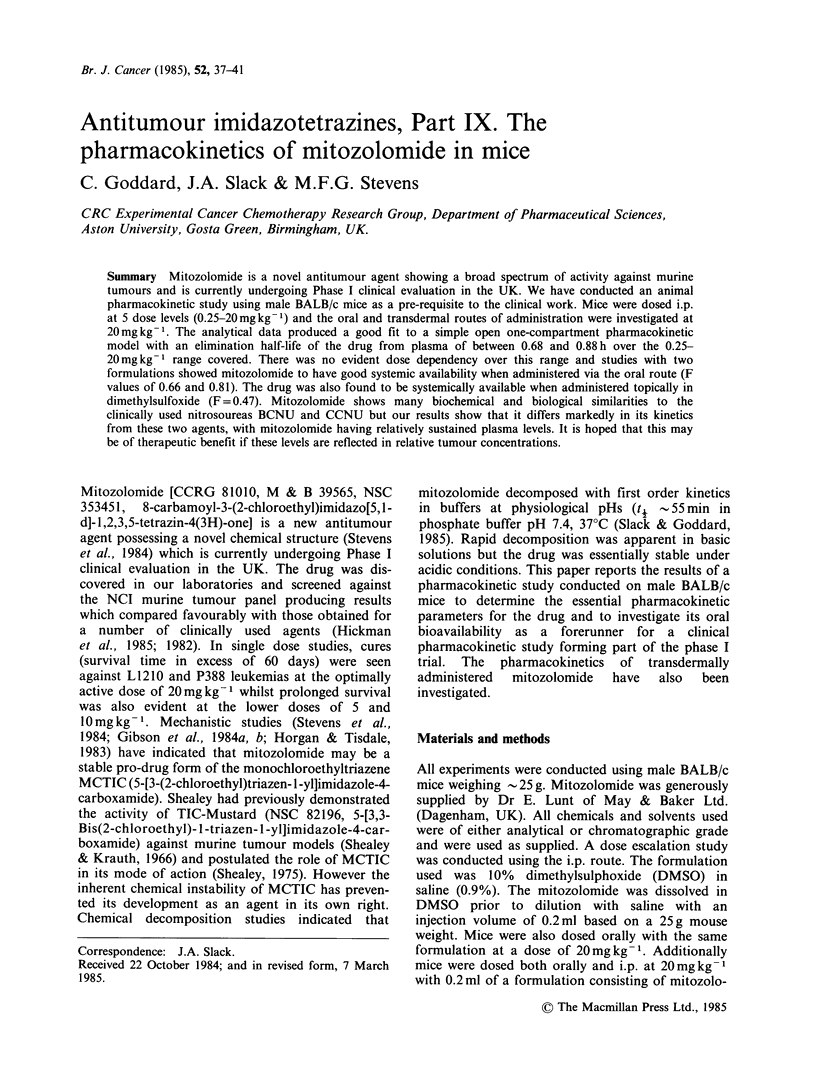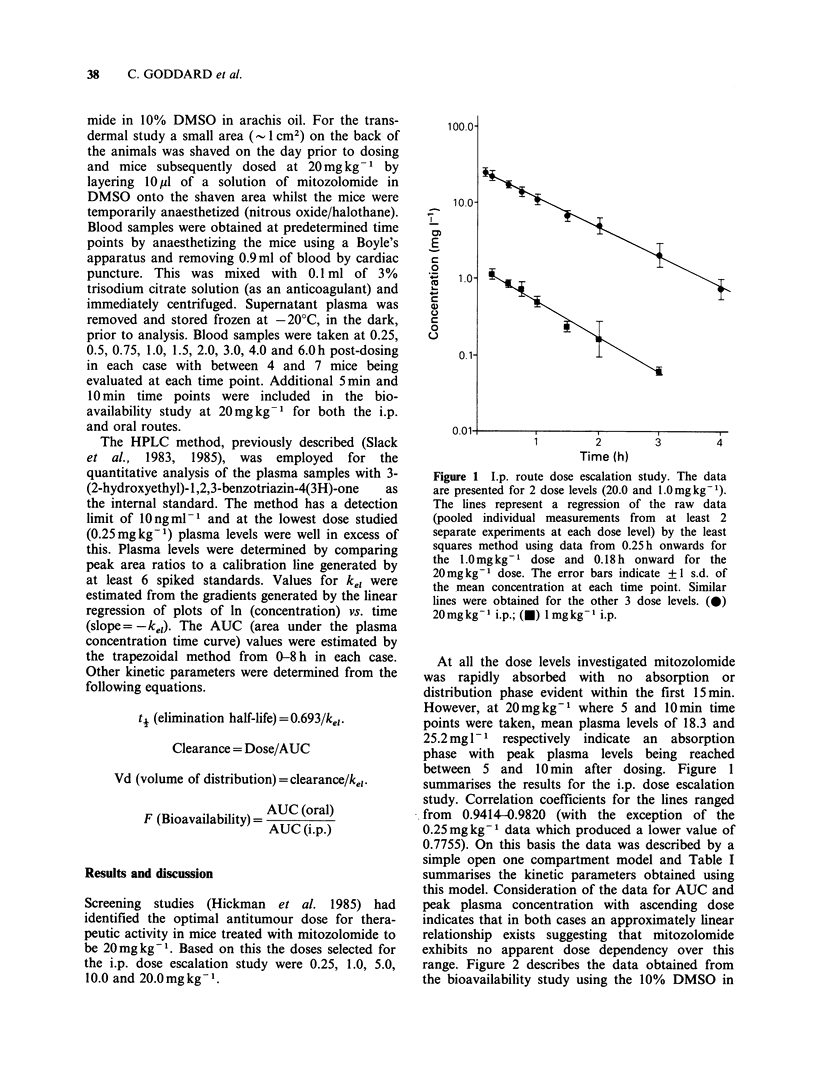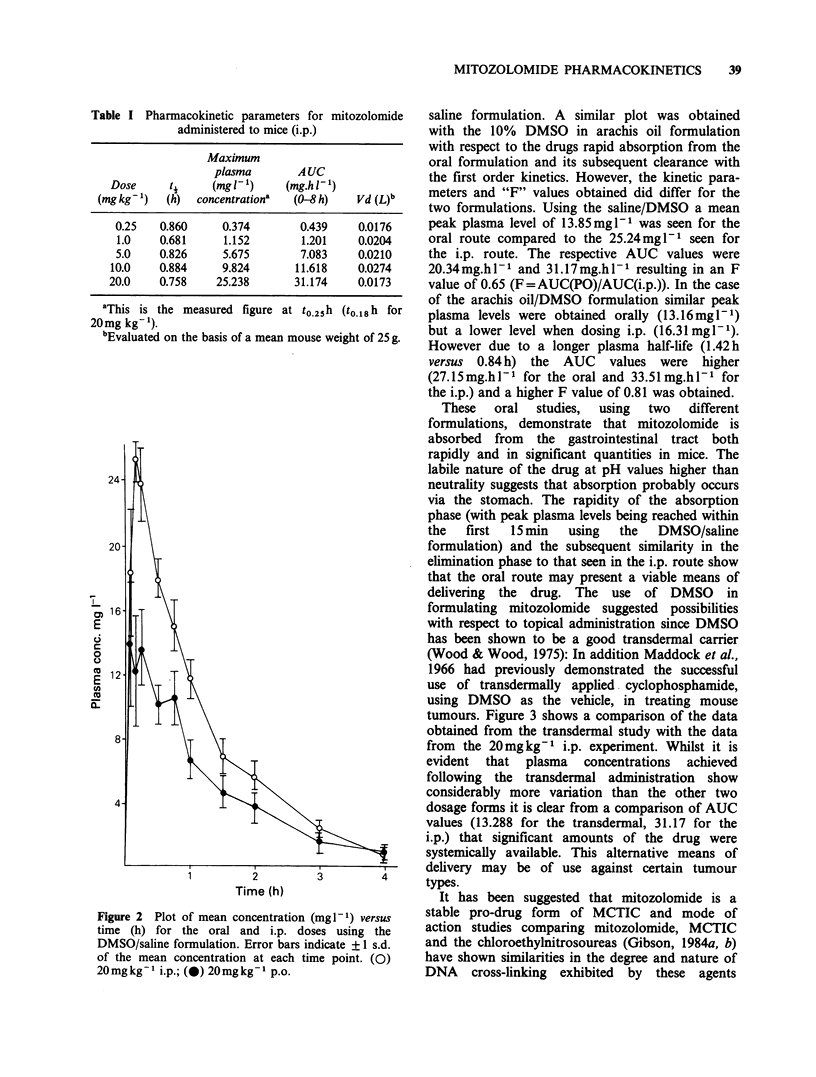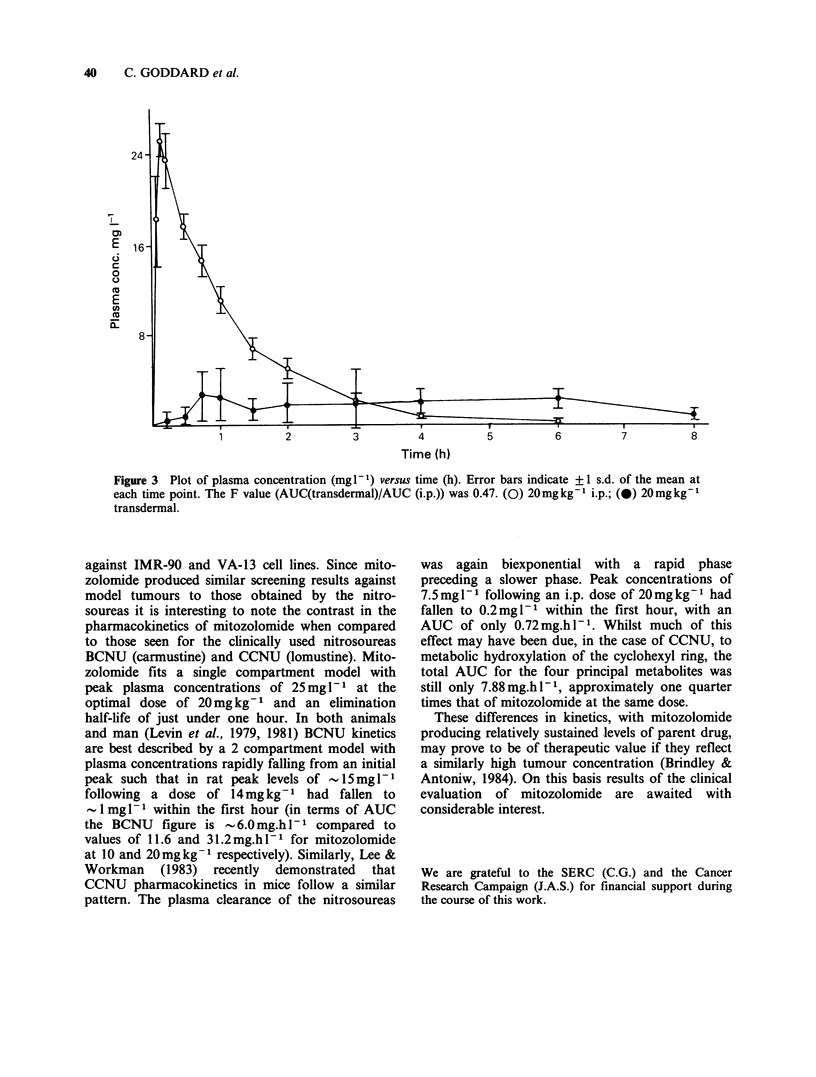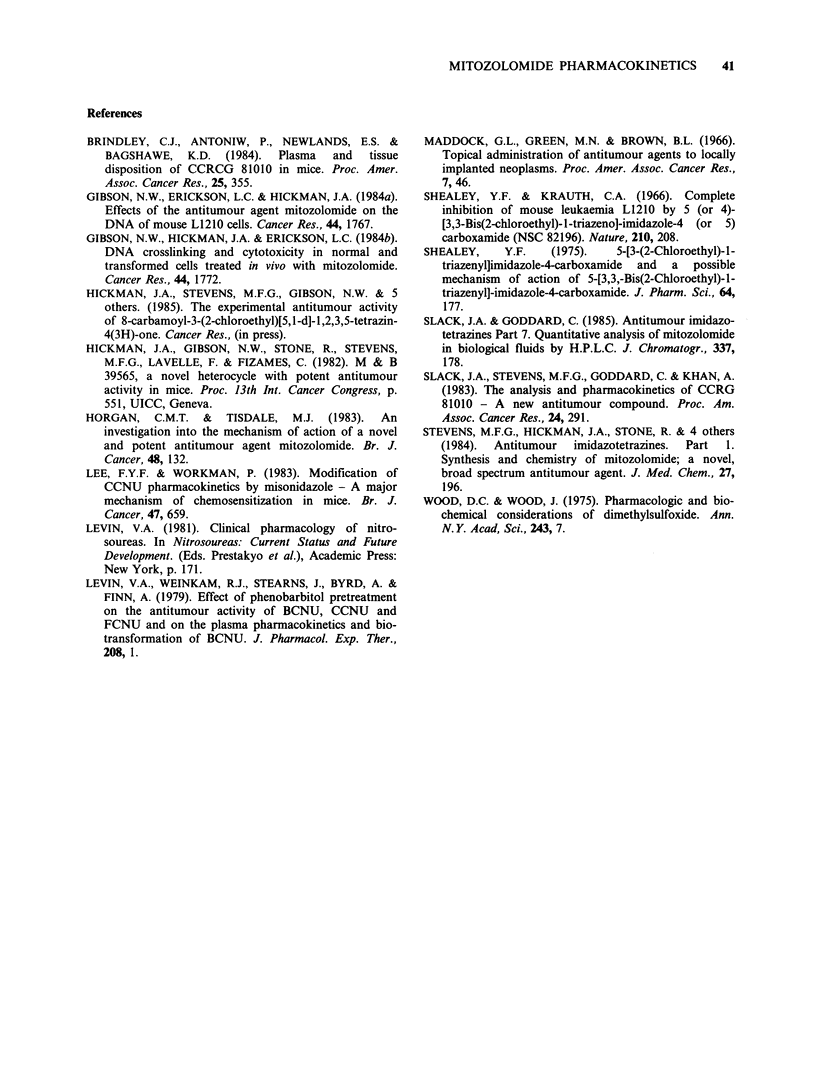# Antitumour imidazotetrazines, Part IX. The pharmacokinetics of mitozolomide in mice.

**DOI:** 10.1038/bjc.1985.145

**Published:** 1985-07

**Authors:** C. Goddard, J. A. Slack, M. F. Stevens

## Abstract

Mitozolomide is a novel antitumour agent showing a broad spectrum of activity against murine tumours and is currently undergoing Phase I clinical evaluation in the UK. We have conducted an animal pharmacokinetic study using male BALB/c mice as a pre-requisite to the clinical work. Mice were dosed i.p. at 5 dose levels (0.25-20 mg kg-1) and the oral and transdermal routes of administration were investigated at 20 mg kg-1. The analytical data produced a good fit to a simple open one-compartment pharmacokinetic model with an elimination half-life of the drug from plasma of between 0.68 and 0.88 h over the 0.25-20 mg kg-1 range covered. There was no evident dose dependency over this range and studies with two formulations showed mitozolomide to have good systemic availability when administered via the oral route (F values of 0.66 and 0.81). The drug was also found to be systemically available when administered topically in dimethylsulfoxide (F = 0.47). Mitozolomide shows many biochemical and biological similarities to the clinically used nitrosoureas BCNU and CCNU but our results show that it differs markedly in its kinetics from these two agents, with mitozolomide having relatively sustained plasma levels. It is hoped that this may be of therapeutic benefit if these levels are reflected in relative tumour concentrations.


*Br. J. Cancer* (1985), **52**, 37–41

### Antitumour imidazotetrazines, Part IX. The pharmacokinetics of mitozolomide in mice

C. Goddard, J.A. Slack & M.F.G. Stevens

*CRC Experimental Cancer Chemotherapy Research Group, Department of Pharmaceutical Sciences, Aston University, Gosta Green, Birmingham, UK.*

**Summary** Mitozolomide is a novel antitumour agent showing a broad spectrum of activity against murine tumours and is currently undergoing Phase I clinical evaluation in the UK. We have conducted an animal pharmacokinetic study using male BALB/c mice as a pre-requisite to the clinical work. Mice were dosed i.p. at 5 dose levels (0.25–20 mg kg$^{-1}$) and the oral and transdermal routes of administration were investigated at 20 mg kg$^{-1}$. The analytical data produced a good fit to a simple open one-compartment pharmacokinetic model with an elimination half-life of the drug from plasma of between 0.68 and 0.88 h over the 0.25–20 mg kg$^{-1}$ range covered. There was no evident dose dependency over this range and studies with two formulations showed mitozolomide to have good systemic availability when administered via the oral route (F values of 0.66 and 0.81). The drug was also found to be systemically available when administered topically in dimethylsulfoxide (F = 0.47). Mitozolomide shows many biochemical and biological similarities to the clinically used nitrosoureas BCNU and CCNU but our results show that it differs markedly in its kinetics from these two agents, with mitozolomide having relatively sustained plasma levels. It is hoped that this may be of therapeutic benefit if these levels are reflected in relative tumour concentrations.

Mitozolomide [CCRG 81010, M & B 39565, NSC 353451, 8-carbamoyl-3-(2-chloroethyl)imidazo[5,1-d]-1,2,3,5-tetrazin-4(3H)-one] is a new antitumour agent possessing a novel chemical structure (Stevens *et al.*, 1984) which is currently undergoing Phase I clinical evaluation in the UK. The drug was discovered in our laboratories and screened against the NCI murine tumour panel producing results which compared favourably with those obtained for a number of clinically used agents (Hickman *et al.*, 1985; 1982). In single dose studies, cures (survival time in excess of 60 days) were seen against L1210 and P388 leukemias at the optimally active dose of 20 mg kg$^{-1}$ whilst prolonged survival was also evident at the lower doses of 5 and 10 mg kg$^{-1}$. Mechanistic studies (Stevens *et al.*, 1984; Gibson *et al.*, 1984*a*, *b*; Horgan & Tisdale, 1983) have indicated that mitozolomide may be a stable pro-drug form of the monochloroethyltriazene MCTIC (5-[3-(2-chloroethyl)triazen-1-yl]imidazole-4-carboxamide). Shealey had previously demonstrated the activity of TIC-Mustard (NSC 82196, 5-[3,3-Bis(2-chloroethyl)-1-triazen-1-yl]imidazole-4-carboxamide) against murine tumour models (Shealey & Krauth, 1966) and postulated the role of MCTIC in its mode of action (Shealey, 1975). However the inherent chemical instability of MCTIC has prevented its development as an agent in its own right. Chemical decomposition studies indicated that mitozolomide decomposed with first order kinetics in buffers at physiological pHs ($t_{\frac{1}{2}}$ ~55 min in phosphate buffer pH 7.4, 37°C (Slack & Goddard, 1985). Rapid decomposition was apparent in basic solutions but the drug was essentially stable under acidic conditions. This paper reports the results of a pharmacokinetic study conducted on male BALB/c mice to determine the essential pharmacokinetic parameters for the drug and to investigate its oral bioavailability as a forerunner for a clinical pharmacokinetic study forming part of the phase I trial. The pharmacokinetics of transdermally administered mitozolomide have also been investigated.

### Materials and methods

All experiments were conducted using male BALB/c mice weighing ~25 g. Mitozolomide was generously supplied by Dr E. Lunt of May & Baker Ltd. (Dagenham, UK). All chemicals and solvents used were of either analytical or chromatographic grade and were used as supplied. A dose escalation study was conducted using the i.p. route. The formulation used was 10% dimethylsulphoxide (DMSO) in saline (0.9%). The mitozolomide was dissolved in DMSO prior to dilution with saline with an injection volume of 0.2 ml based on a 25 g mouse weight. Mice were also dosed orally with the same formulation at a dose of 20 mg kg$^{-1}$. Additionally mice were dosed both orally and i.p. at 20 mg kg$^{-1}$ with 0.2 ml of a formulation consisting of mitozolo-

Correspondence: J.A. Slack.
Received 22 October 1984; and in revised form, 7 March 1985.

© The Macmillan Press Ltd., 1985

mide in 10% DMSO in arachis oil. For the transdermal study a small area (~1 cm$^2$) on the back of the animals was shaved on the day prior to dosing and mice subsequently dosed at 20 mg kg$^{-1}$ by layering 10 $\mu$l of a solution of mitozolomide in DMSO onto the shaven area whilst the mice were temporarily anaesthetized (nitrous oxide/halothane). Blood samples were obtained at predetermined time points by anaesthetizing the mice using a Boyle's apparatus and removing 0.9 ml of blood by cardiac puncture. This was mixed with 0.1 ml of 3% trisodium citrate solution (as an anticoagulant) and immediately centrifuged. Supernatant plasma was removed and stored frozen at −20°C, in the dark, prior to analysis. Blood samples were taken at 0.25, 0.5, 0.75, 1.0, 1.5, 2.0, 3.0, 4.0 and 6.0 h post-dosing in each case with between 4 and 7 mice being evaluated at each time point. Additional 5 min and 10 min time points were included in the bioavailability study at 20 mg kg$^{-1}$ for both the i.p. and oral routes.

The HPLC method, previously described (Slack *et al.*, 1983, 1985), was employed for the quantitative analysis of the plasma samples with 3-(2-hydroxyethyl)-1,2,3-benzotriazin-4(3H)-one as the internal standard. The method has a detection limit of 10 ng ml$^{-1}$ and at the lowest dose studied (0.25 mg kg$^{-1}$) plasma levels were well in excess of this. Plasma levels were determined by comparing peak area ratios to a calibration line generated by at least 6 spiked standards. Values for $k_{el}$ were estimated from the gradients generated by the linear regression of plots of ln (concentration) *vs.* time (slope = $-k_{el}$). The AUC (area under the plasma concentration time curve) values were estimated by the trapezoidal method from 0–8 h in each case. Other kinetic parameters were determined from the following equations.

$$t_{\frac{1}{2}} \text{ (elimination half-life)} = 0.693/k_{el}.$$

$$\text{Clearance} = \text{Dose/AUC}$$

$$\text{Vd (volume of distribution)} = \text{clearance}/k_{el}.$$

$$F \text{ (Bioavailability)} = \frac{\text{AUC (oral)}}{\text{AUC (i.p.)}}$$

### Results and discussion

Screening studies (Hickman *et al.* 1985) had identified the optimal antitumour dose for therapeutic activity in mice treated with mitozolomide to be 20 mg kg$^{-1}$. Based on this the doses selected for the i.p. dose escalation study were 0.25, 1.0, 5.0, 10.0 and 20.0 mg kg$^{-1}$.

**Figure 1** I.p. route dose escalation study. The data are presented for 2 dose levels (20.0 and 1.0 mg kg$^{-1}$). The lines represent a regression of the raw data (pooled individual measurements from at least 2 separate experiments at each dose level) by the least squares method using data from 0.25 h onwards for the 1.0 mg kg$^{-1}$ dose and 0.18 h onward for the 20 mg kg$^{-1}$ dose. The error bars indicate ±1 s.d. of the mean concentration at each time point. Similar lines were obtained for the other 3 dose levels. (●) 20 mg kg$^{-1}$ i.p.; (■) 1 mg kg$^{-1}$ i.p.

At all the dose levels investigated mitozolomide was rapidly absorbed with no absorption or distribution phase evident within the first 15 min. However, at 20 mg kg$^{-1}$ where 5 and 10 min time points were taken, mean plasma levels of 18.3 and 25.2 mg l$^{-1}$ respectively indicate an absorption phase with peak plasma levels being reached between 5 and 10 min after dosing. Figure 1 summarises the results for the i.p. dose escalation study. Correlation coefficients for the lines ranged from 0.9414–0.9820 (with the exception of the 0.25 mg kg$^{-1}$ data which produced a lower value of 0.7755). On this basis the data was described by a simple open one compartment model and Table I summarises the kinetic parameters obtained using this model. Consideration of the data for AUC and peak plasma concentration with ascending dose indicates that in both cases an approximately linear relationship exists suggesting that mitozolomide exhibits no apparent dose dependency over this range. Figure 2 describes the data obtained from the bioavailability study using the 10% DMSO in

**Table I** Pharmacokinetic parameters for mitozolomide administered to mice (i.p.)

| *Dose* (*mg kg*$^{-1}$) | $t_{\frac{1}{2}}$ (*h*) | *Maximum plasma* (*mg l*$^{-1}$) *concentration*[a] | *AUC* (*mg.h l*$^{-1}$) (*0–8 h*) | *Vd (L)*[b] |
|---|---|---|---|---|
| 0.25 | 0.860 | 0.374 | 0.439 | 0.0176 |
| 1.0 | 0.681 | 1.152 | 1.201 | 0.0204 |
| 5.0 | 0.826 | 5.675 | 7.083 | 0.0210 |
| 10.0 | 0.884 | 9.824 | 11.618 | 0.0274 |
| 20.0 | 0.758 | 25.238 | 31.174 | 0.0173 |

[a]This is the measured figure at $t_{0.25}$h ($t_{0.18}$h for 20 mg kg$^{-1}$).
[b]Evaluated on the basis of a mean mouse weight of 25 g.

**Figure 2** Plot of mean concentration (mg l$^{-1}$) *versus* time (h) for the oral and i.p. doses using the DMSO/saline formulation. Error bars indicate ±1 s.d. of the mean concentration at each time point. (○) 20 mg kg$^{-1}$ i.p.; (●) 20 mg kg$^{-1}$ p.o.

saline formulation. A similar plot was obtained with the 10% DMSO in arachis oil formulation with respect to the drugs rapid absorption from the oral formulation and its subsequent clearance with the first order kinetics. However, the kinetic parameters and "F" values obtained did differ for the two formulations. Using the saline/DMSO a mean peak plasma level of 13.85 mg l$^{-1}$ was seen for the oral route compared to the 25.24 mg l$^{-1}$ seen for the i.p. route. The respective AUC values were 20.34 mg.h l$^{-1}$ and 31.17 mg.h l$^{-1}$ resulting in an F value of 0.65 (F = AUC(PO)/AUC(i.p.)). In the case of the arachis oil/DMSO formulation similar peak plasma levels were obtained orally (13.16 mg l$^{-1}$) but a lower level when dosing i.p. (16.31 mg l$^{-1}$). However due to a longer plasma half-life (1.42 h *versus* 0.84 h) the AUC values were higher (27.15 mg.h l$^{-1}$ for the oral and 33.51 mg.h l$^{-1}$ for the i.p.) and a higher F value of 0.81 was obtained.

These oral studies, using two different formulations, demonstrate that mitozolomide is absorbed from the gastrointestinal tract both rapidly and in significant quantities in mice. The labile nature of the drug at pH values higher than neutrality suggests that absorption probably occurs via the stomach. The rapidity of the absorption phase (with peak plasma levels being reached within the first 15 min using the DMSO/saline formulation) and the subsequent similarity in the elimination phase to that seen in the i.p. route show that the oral route may present a viable means of delivering the drug. The use of DMSO in formulating mitozolomide suggested possibilities with respect to topical administration since DMSO has been shown to be a good transdermal carrier (Wood & Wood, 1975): In addition Maddock *et al.*, 1966 had previously demonstrated the successful use of transdermally applied cyclophosphamide, using DMSO as the vehicle, in treating mouse tumours. Figure 3 shows a comparison of the data obtained from the transdermal study with the data from the 20 mg kg$^{-1}$ i.p. experiment. Whilst it is evident that plasma concentrations achieved following the transdermal administration show considerably more variation than the other two dosage forms it is clear from a comparison of AUC values (13.288 for the transdermal, 31.17 for the i.p.) that significant amounts of the drug were systemically available. This alternative means of delivery may be of use against certain tumour types.

It has been suggested that mitozolomide is a stable pro-drug form of MCTIC and mode of action studies comparing mitozolomide, MCTIC and the chloroethylnitrosoureas (Gibson, 1984*a*, *b*) have shown similarities in the degree and nature of DNA cross-linking exhibited by these agents

**Figure 3** Plot of plasma concentration (mg $l^{-1}$) *versus* time (h). Error bars indicate $\pm 1$ s.d. of the mean at each time point. The F value (AUC(transdermal)/AUC (i.p.)) was 0.47. (○) 20 mg $kg^{-1}$ i.p.; (●) 20 mg $kg^{-1}$ transdermal.

against IMR-90 and VA-13 cell lines. Since mitozolomide produced similar screening results against model tumours to those obtained by the nitrosoureas it is interesting to note the contrast in the pharmacokinetics of mitozolomide when compared to those seen for the clinically used nitrosoureas BCNU (carmustine) and CCNU (lomustine). Mitozolomide fits a single compartment model with peak plasma concentrations of 25 mg $l^{-1}$ at the optimal dose of 20 mg $kg^{-1}$ and an elimination half-life of just under one hour. In both animals and man (Levin *et al.*, 1979, 1981) BCNU kinetics are best described by a 2 compartment model with plasma concentrations rapidly falling from an initial peak such that in rat peak levels of ~15 mg $l^{-1}$ following a dose of 14 mg $kg^{-1}$ had fallen to ~1 mg $l^{-1}$ within the first hour (in terms of AUC the BCNU figure is ~6.0 mg.h $l^{-1}$ compared to values of 11.6 and 31.2 mg.h $l^{-1}$ for mitozolomide at 10 and 20 mg $kg^{-1}$ respectively). Similarly, Lee & Workman (1983) recently demonstrated that CCNU pharmacokinetics in mice follow a similar pattern. The plasma clearance of the nitrosoureas was again biexponential with a rapid phase preceding a slower phase. Peak concentrations of 7.5 mg $l^{-1}$ following an i.p. dose of 20 mg $kg^{-1}$ had fallen to 0.2 mg $l^{-1}$ within the first hour, with an AUC of only 0.72 mg.h $l^{-1}$. Whilst much of this effect may have been due, in the case of CCNU, to metabolic hydroxylation of the cyclohexyl ring, the total AUC for the four principal metabolites was still only 7.88 mg.h $l^{-1}$, approximately one quarter times that of mitozolomide at the same dose.

These differences in kinetics, with mitozolomide producing relatively sustained levels of parent drug, may prove to be of therapeutic value if they reflect a similarly high tumour concentration (Brindley & Antoniw, 1984). On this basis results of the clinical evaluation of mitozolomide are awaited with considerable interest.

We are grateful to the SERC (C.G.) and the Cancer Research Campaign (J.A.S.) for financial support during the course of this work.

### References

BRINDLEY, C.J., ANTONIW, P., NEWLANDS, E.S. & BAGSHAWE, K.D. (1984). Plasma and tissue disposition of CCRCG 81010 in mice. *Proc. Amer. Assoc. Cancer Res.*, **25**, 355.

GIBSON, N.W., ERICKSON, L.C. & HICKMAN, J.A. (1984*a*). Effects of the antitumour agent mitozolomide on the DNA of mouse L1210 cells. *Cancer Res.*, **44**, 1767.

GIBSON, N.W., HICKMAN, J.A. & ERICKSON, L.C. (1984*b*). DNA crosslinking and cytotoxicity in normal and transformed cells treated *in vivo* with mitozolomide. *Cancer Res.*, **44**, 1772.

HICKMAN, J.A., STEVENS, M.F.G., GIBSON, N.W. & 5 others. (1985). The experimental antitumour activity of 8-carbamoyl-3-(2-chloroethyl)[5,1-d]-1,2,3,5-tetrazin-4(3H)-one. *Cancer Res.*, (in press).

HICKMAN, J.A., GIBSON, N.W., STONE, R., STEVENS, M.F.G., LAVELLE, F. & FIZAMES, C. (1982). M & B 39565, a novel heterocycle with potent antitumour activity in mice. *Proc. 13th Int. Cancer Congress*, p. 551, UICC, Geneva.

HORGAN, C.M.T. & TISDALE, M.J. (1983). An investigation into the mechanism of action of a novel and potent antitumour agent mitozolomide. *Br. J. Cancer*, **48**, 132.

LEE, F.Y.F. & WORKMAN, P. (1983). Modification of CCNU pharmacokinetics by misonidazole – A major mechanism of chemosensitization in mice. *Br. J. Cancer*, **47**, 659.

LEVIN, V.A. (1981). Clinical pharmacology of nitrosoureas. In *Nitrosoureas: Current Status and Future Development*. (Eds. Prestakyo *et al.*), Academic Press: New York, p. 171.

LEVIN, V.A., WEINKAM, R.J., STEARNS, J., BYRD, A. & FINN, A. (1979). Effect of phenobarbitol pretreatment on the antitumour activity of BCNU, CCNU and FCNU and on the plasma pharmacokinetics and biotransformation of BCNU. *J. Pharmacol. Exp. Ther.*, **208**, 1.

MADDOCK, G.L., GREEN, M.N. & BROWN, B.L. (1966). Topical administration of antitumour agents to locally implanted neoplasms. *Proc. Amer. Assoc. Cancer Res.*, **7**, 46.

SHEALEY, Y.F. & KRAUTH, C.A. (1966). Complete inhibition of mouse leukaemia L1210 by 5 (or 4)-[3,3-Bis(2-chloroethyl)-1-triazeno]-imidazole-4 (or 5) carboxamide (NSC 82196). *Nature*, **210**, 208.

SHEALEY, Y.F. (1975). 5-[3-(2-Chloroethyl)-1-triazenyl]imidazole-4-carboxamide and a possible mechanism of action of 5-[3,3,-Bis(2-Chloroethyl)-1-triazenyl]-imidazole-4-carboxamide. *J. Pharm. Sci.*, **64**, 177.

SLACK, J.A. & GODDARD, C. (1985). Antitumour imidazotetrazines Part 7. Quantitative analysis of mitozolomide in biological fluids by H.P.L.C. *J. Chromatogr.*, **337**, 178.

SLACK, J.A., STEVENS, M.F.G., GODDARD, C. & KHAN, A. (1983). The analysis and pharmacokinetics of CCRG 81010 – A new antitumour compound. *Proc. Am. Assoc. Cancer Res.*, **24**, 291.

STEVENS, M.F.G., HICKMAN, J.A., STONE, R. & 4 others (1984). Antitumour imidazotetrazines. Part 1. Synthesis and chemistry of mitozolomide; a novel, broad spectrum antitumour agent. *J. Med. Chem.*, **27**, 196.

WOOD, D.C. & WOOD, J. (1975). Pharmacologic and biochemical considerations of dimethylsulfoxide. *Ann. N.Y. Acad, Sci.*, **243**, 7.